# Alterations of panoramic radiomorphometric indices in children and adolescents with beta-thalassemia major: A fractal analysis study

**DOI:** 10.4317/medoral.24784

**Published:** 2021-12-07

**Authors:** Burcu Yagmur, Humeyra Tercanli-Alkis, Funda Tayfun-Kupesiz, Huseyin Karayilmaz, Osman Alphan Kupesiz

**Affiliations:** 1 PhD. Department of Pedodontics, Faculty of Dentistry, University of Akdeniz, Antalya, Turkey; 2 PhD. Department of Oral and Maxillofacial Radiology, Faculty of Dentistry, University of Akdeniz, Antalya, Turkey; 3 Assistant professor. Department of Pediatric Hematology and Oncology, Faculty of Medicine, University of Akdeniz, Antalya, Turkey; 4 Professor. Department of Pedodontics, Faculty of Dentistry, University of Akdeniz, Antalya, Turkey; 5 Professor. Department of Pediatric Hematology and Oncology, Faculty of Medicine, University of Akdeniz, Antalya, Turkey

## Abstract

**Background:**

Beta-thalassemia major is an inherited disorder that can cause bone deformity and loss of bone mineral density. The objective of this study is to evaluate the cortical and trabecular mandibular bone morphology of children and adolescents who have beta-thalassemia major (ß-TM) using a fractal dimension (FD) analysis and different panoramic radiomorphometric indices with digital panoramic radiographic images (DPRIs).

**Material and Methods:**

The study included 80 patients (with 40 patients each of ß-TM and control). The mandibular cortical width (MCW), panoramic mandibular index (PMI), mandibular cortical index (MCI), and simple visual estimation (SVE) were evaluated, and an FD analysis of five regions of interest (ROIs) (ROI 1: in basal cortical bone; ROI 2: in premolar region; ROI 3: in molar region; ROI 4: in angulus mandible and ROI 5: in condyle region) was obtained in all DPRIs. Quantitative variables were analyzed using the student’s t-test , Kruskal–Wallis and Mann-Whitney U tests.

**Results:**

When the ß-TM groups were compared with controls, there were no statistically significant differences found in the mean FD values, the ROIs of the trabecular bone, or the SVE. There was a significant correlation in the mean MCW, PMI, ROI of cortical bone (ROI 1), and MCI between ß-TM and control groups (*p* < 0.001, *p* < 0.001, *p* = 0.047, and *p* = 0.046, respectively). The mean MCW values correlated with the SVE in both the ß-TM and control groups (*p* = 0.031 and *p* < 0.001, respectively). While the mean MCW values correlated with the MCI (*p* = 0.04) in the control group, the mean MCW values were not correlated with the MCI (*p* = 0.493) in ß-TM group.

**Conclusions:**

The current study revealed lower MCW and PMI values in the ß-TM group. While the mean FD values of trabecular bone is similar to the control groups, the mean FD value is lower in cortical bone in the ß-TM group. MCW, PMI, FD of cortical bone and MCI may be key indicators in individuals with beta-thalassemia major.

** Key words:**Beta-thalassemia major, fractals, panoramic radiography.

## Introduction

Beta-thalassemia major (ß-TM), also known as Cooley' s anemia or Mediterranean anemia, is an inherited disorder of beta globin chain synthesis, which leads to ineffective erythropoiesis and requires chronic blood transfusion therapy (1). This disease causes iron overload, marrow expansion, delayed puberty, and hormonal complications, resulting in bone deformity and loss of bone mineral density (BMD) (2). The common orofacial features contain frontal bossing, skeletal changes because of bony overgrowth (with typical appearances known as chipmunk faces), upper lip retraction, and the protrusion of premaxilla bone associated with alveolar enlargement (3). This disease leads to an increased prevalence of non-traumatic fractures, both long bone and vertebral (2,4).

Little is known about the prevalence of low bone mass or the current rate of fractures and associations between bone mass and bone pain in children and adolescents with ß-TM (5). Dual energy X-ray absorptiometry (DEXA) is the most common method used to assess bone health in children and adolescents (6).

On the other hand, panoramic radiographic images can show the loss of BMD (7,8). To determine this, different panoramic radiomorphometric indices, such as mandibular cortical width (MCW), panoramic mandibular index (PMI), mandibular cortical index (MCI), and simple visual estimation (SVE), have been used in the literature (7-12). Fractal dimension (FD), a statistical tissue analysis, can reflect mineral loss in the bone in radiographic images (13). Although the mandibular bone structure in patients with osteoporosis has been examined with FD (13,14), very few studies investigate the mandibular bone morphology in TM patients with FD (13,15,16).

This study hypothesizes that children and adolescents with ß-TM may have different bone geometries because of an increased risk of bone fractures. The aim of this retrospective study is to evaluate the cortical and trabecular mandibular bone morphology of children and adolescents with ß-TM and a systemically healthy group using FD analysis and different panoramic radiomorphometric indices by means of digital panoramic radiographic images (DPRIs).

## Material and Methods

- Study design

This retrospective study was conducted in the Department of Pedodontics, Faculty of Dentistry, Akdeniz University, in the west Mediterranean region of Turkey, in Antalya. The records of patients, who presented to the Department of Pedodontics, Faculty of Dentistry, Akdeniz University between 2012 and 2019 for different purposes, were assessed, and 53 ß-TM patients were detected. The DPRIs of the 53 patients were retrospectively obtained from the Department of Oral and Maxillofacial Radiology, Akdeniz University, and the following exclusion criteria were applied: (1) those who had other systemic diseases in addition to ß-TM; (2) those who regularly had blood transfusion in Akdeniz University, Faculty of Medicine, Department of Pediatric Hematology-Oncology; (3) those in whom temporomandibular joint pathology was suspected; (4) image quality was poor and had horizontal and vertical distortions; (5) DPRI which had sclerotic area in mandibula; (6) DPRI with radiologically periodontally unhealthy tissues; (7) DPRIs in which the mental foramen cannot be clearly visualized, and (8) DPRIs in which the regions of interest (ROIs) for FD analysis cannot be clearly visualized and images that had anatomical superposition to these areas.

When these exclusion criteria were taken into consideration for the 53 patients, the radiographic images of 13 patients with mental foramen could not be clearly visualized and were excluded from the study, thus 40 patients were finally included in the study for the ß-TM group. For the control group, an equal number of patients to the ß-TM group (included same age range and similar gender distribution) without any systemic diseases were included (n = 40). The anamnesis data of the patients were detected using the Metasoft Dentasist Program (version 3.0.448, Eskisehir, Turkey).

The DPRIs were obtained by the same X-ray technician using a Planmeca ProMax panoramic device (Planmeca Oy, Helsinki, Finland) in accordance with the manufacturer’s instructions (voltage, 64 kVp; tube current, 7 mA; exposure time, 16 s). The DPRIs were evaluated using the same LED monitor and approximately 40–50 cm away from the LED monitor by the same investigator, who is a dental radiology expert. The evaluation was done in a reduced-light room with tonal adjustments made on images to maximize the view. Only ten panoramic images were evaluated per day with about one-hour breaks every five images to prevent investigator fatigue. Measurements were automatically calibrated with the Planmeca Romexis 4.0 software program developed for the Planmeca ProMax device (Planmeca Oy, 00880 Helsinki, Finland) as per the manufacturer’s instructions.

- Radiomorphometric indices

**Figure 1:**
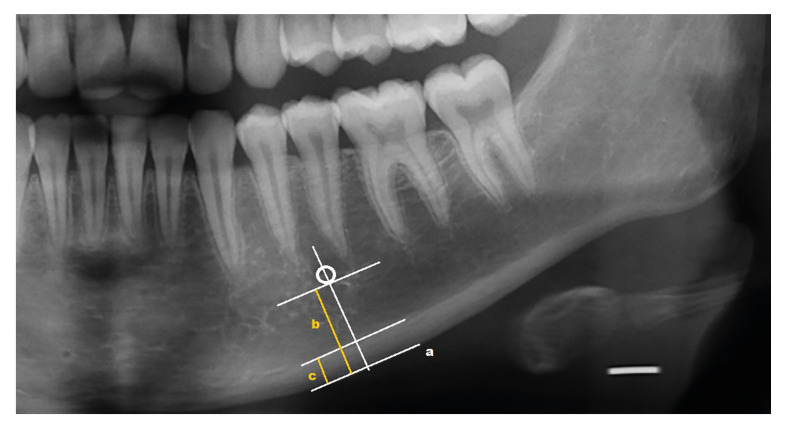
A line parallel to the inferior border of the mandible (a); distance between the inferior border of the mental foramen and "a" line (b); mandibular cortical width (c); and panoramic mandibular index ( c/b).

Radiomorphometric indices were evaluated as follows:

MCW: The mental foramen was detected on the DPRI, and two lines were drawn tangent to the lower border of the mandible and parallel to the upper border of the mandibular cortical layer. Then the center of the mental foramen and the lower border of the mandible were joined with a vertical line. The distance between the two parallel lines was measured separately on the right and left sides (9) (Fig. 1), and the mean values were calculated as the MCW.

PMI: The ratio of the MCW to the distance from the lower border of the mandible to the inferior edge of the mental foramen was calculated separately on the right and left sides, and the mean values were calculated as the PMI (10) (Fig. 1).

MCI: This was evaluated based on the morphologic changes in the cortical bone at the mandibular base as follows:

C1: endosteal margins of the cortex are equal and sharp on the right and left sides.

C2: endosteal margins show defects in the form of semilunar (lacunar resorption) and/or endosteal cortical residues on one or both sides.

**Figure 2:**
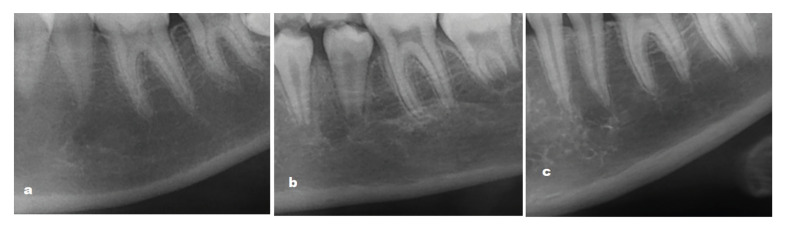
Classification of mandibular cortical index. C1: endosteal margins of the cortex are sharp and equal on both sides (a); C2: endosteal margins show defects in the form of semiulnar (lacunar resorption), and / or endosteal cortical residues on one or both sides (b); C3: the cortical layer contains heavy endosteal cortical residues and is clearly porous (c).

C3: the cortical layer is clearly porous and contains heavy endosteal cortical residues (7) (Fig. 2).

**Figure 3:**
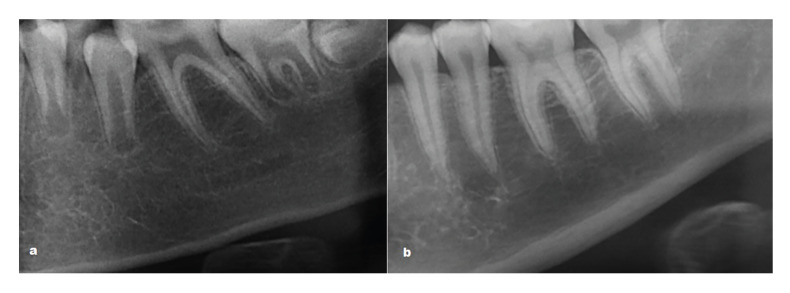
Classification of simple visual estimation: thin (a); not thin (b).

SVE: The cortex was classified into two categories based on the SVE of the mandibular inferior cortex widths according to observer experience: thin and not thin (12) (Fig. 3).

- Fractal dimension analysis (FDA)

**Figure 4:**
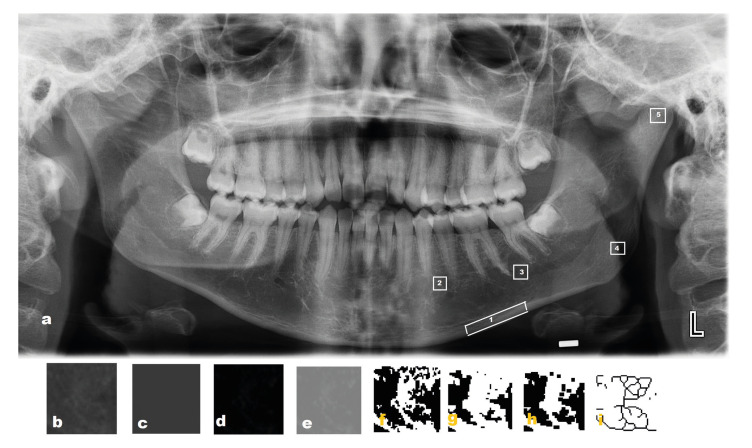
Panoramic radiograph with the five selected interest areas; 1: interest area on the cortical bone, 2: interest area of the premolar region, 3: interest area of molar region, 4: interest area of the angulus mandible, 5: interest area of condyle (a); cropped and duplicated image (b); a gaussian blurred image (c); a subtraction image (d); an added 128 image (e); binarization (f); erosion (g); dilation (h); and skeletonization (i).

The FDA for each image was conducted following White and Rudolph (17) using the box counting method. The ImageJ version 1.3 software program (National Institutes of Health, Bethesda, MD), which can be downloaded from the “http://rsb.info.nih.gov/ij/download.html” website, was used to analyze the images. Five ROIs from the left side of the mandible were determined for the FDA: ROI 1: a rectangle in the basal cortical bone, distal to the mental foramen extending to the distal root of the first permanent molar (11); ROI 2:64*64 pixel square in the premolar region (18); ROI 3:64*64 pixel square in the molar region(18);ROI 4: 50x50 pixel square in the geometric center of the angulus mandible; and ROI 5: 64*64 pixel square in the geometric center of the condyle (15) (Fig. 4). For the FDA, DPRIs were saved in TIFF format. The ROIs selection was made, and the image was cut and duplicated. Gaussian filter (sigma 35) was applied to the duplicated image, and the blurred image were subtracted from the original image. A gray value of 128 was added to each pixel location. The obtained image was made binary, and with this process, the regions representing the trabecular bone were adjusted to white, and marrow cavities were adjusted to black. The image was eroded and dilated. After dilatation, the image was skeletonized and used for fractal analysis. The FDA was calculated by the box counting method. The width of the square boxes was 2, 3, 4, 6, 8, 12, 16, 32, and 64 pixels. At the end of this process, a single numerical value was obtained (Fig. 4).

After four weeks, MCW, PMI measurements, MCI, SVE, and FDA in 40 randomly selected patients were repeated, and intra-observer variability was assessed.

- Statistical analysis

Data was statistically analyzed using an SPSS software package (version 23.0, SPSS Chicago, USA). Descriptive statistics were presented with mean ± standard deviation and median (minimum-maximum) values. After the homogeneity of variance and normal distribution had been verified through Levene’s test, quantitative variables among the groups were compared using the student’s t-test for parametric data and Kruskal–Wallis and Mann -Whitney U tests were used for non-parametric data. Qualitative variables were examined using the Kruskal–Wallis, Mann -Whitney U, chi-square, and exact Fisher tests, respectively. For numerical data, intra-observer reliability was assessed by the interclass correlation coefficient (ICC), and for nominal data, the kappa coefficient was used. Statistical significance was accepted at *p* < 0.05.

## Results

There were 19 male and 21 female patients in the ß-TM group, and the mean age of the patients was 11.98 ± 2.79 years. There were 18 male and 22 female patients in the control group, and the mean age of the patients was 11.67 ± 2.53 years. There was no relationship between the presence of ß-TM and gender (*p* = 0.823).

The ICC indicated good reliability for each measurement, including MCW (ICC = 0.96), and PMI (ICC = 0.9), and the kappa coefficients were 0.84 and 0.83, respectively, for MCI and SVE, ROI 1 (ICC = 0.97), ROI 2 (ICC = 0.94), ROI 3 (ICC = 0.98), ROI 4 (ICC = 0.98), ROI 5 (ICC = 0.98),

The mean MCW and PMI measurements were lower in the ß-TM group than in the control group. There was a significant relationship between the ß-TM and control groups’ mean MCW measurements (3.69 ± 0.81 and 4.96 ± 1.42, respectively, and *p* < 0.001) and mean PMI measurements (0.24 ± 0.05 and 0.34 ± 0.1, respectively, and *p* < 0.001).

According to the MCI classification, while 25 C1, 10 C2, and 5 C3 patients were found in the ß-TM group, 33 C1, 5 C2, and 2 C3 patients were found in the control group. According to the SVE classification, there were 11 ''thin" and 29 "not thin" patients in the ß-TM group, and there were 14 ''thin" and 26 "not thin" patients in the control group. While there was a significant difference between the ß-TM and control groups according to the MCI (*p* = 0.046), there was no significant difference between the ß-TM and control groups according to the SVE (*p* = 0.472).

In the ß-TM group, while the mean MCW values were correlated with the SVE (*p* = 0.031), the mean PMI values were not the SVE (*p* = 0.303). On the other hand, the mean MCW values and the mean PMI values were not correlated with the MCI (*p* = 0.493 and *p* = 0.809, respectively). The mean MCW values were 3.25 ± 0.44 mm and 3.86 ± 0.86 mm in the "thin" and "not thin" groups, respectively, and the mean PMI values were 0.22 ± 0.04 mm and 0.24 ± 0.05 mm in the "thin" and "not thin" groups, respectively.

In the control group, the mean MCW values and the mean PMI values were correlated with the SVE (*p* < 0.001 and *p* = 0.027, respectively). On the other hand, while the mean MCW values correlated with the MCI (*p* = 0.04), the mean PMI values were not correlated with the MCI (*p* = 0.177). The mean MCW values were 3.85 ± 1.01 mm and 5.55 ± 1.25 mm in the "thin" and "not thin" groups, respectively, and the mean PMI values were 0.29 ± 0.06 mm and 0.36 ± 0.1 mm in the "thin" and "not thin" groups, respectively.

The mean FD values were found nearly equal in the ß-TM group and the control group (1.29 ± 0.06 and 1.3 ± 0.04, respectively), without statistical significance (*p* = 0.334). When ROIs are taken into consideration, mean FD values were not significantly different between regions in the ß-TM and control groups except for ROI 1 (*p* = 0.047). Table 1 shows the minimum, maximum, mean, standard deviation, and *p* values of FD in the ß-TM and control groups.

In the ß-TM group and control group, there was a significant relationship between the mean MCW, the mean PMI values, and the mean FD and mean ROI 1 FD values. Table 2 shows the minimum, maximum, mean, standard deviation, and *p* values of MCW-FD, PMI-FD, MCW-ROI 1 FD, and PMI- ROI 1 for the ß-TM and control groups.

When ROIs are considered in the ß-TM group, there was no significant difference between any ROIs and both the MCI and SVE. In the control group, there was a significant correlation between ROI 4 FD and the SVE (*p* = 0.042), and ROI 5 FD and the MCI (*p* = 0.022).

**Table 1:**
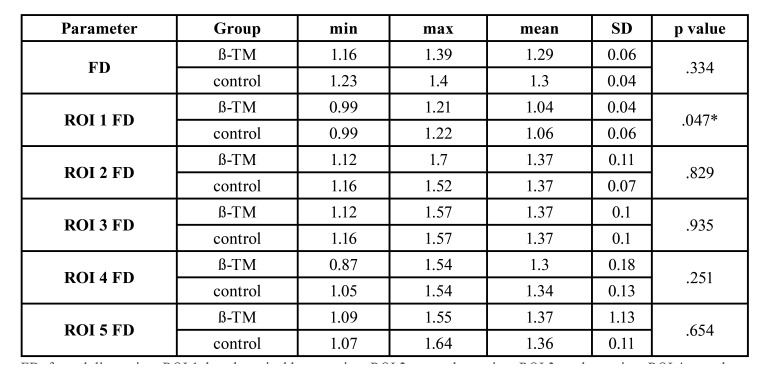
The minimum, maximum, mean, standard deviation and *p* values of FD in all groups.

**Table 2:**
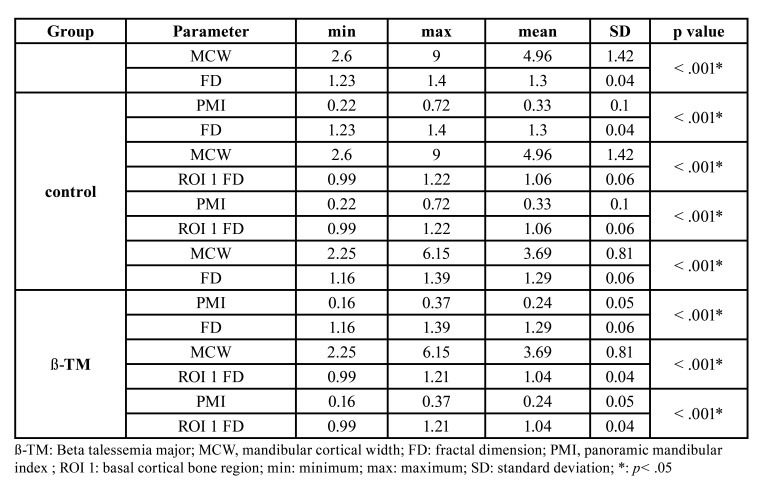
The minimum, maximum, mean, standard deviation and *p* values of MCW- FD, PMI- FD, MCW- ROI 1 FD and PMI- ROI 1 FD in ß-TM and control group.

## Discussion

It has been proven that panoramic radiomorphometric indices can be used as a preliminary diagnosis in evaluating osteoporotic changes and that there is a good correlation between panoramic radiographs and BMD values (15,19). Moreover, in panoramic radiomorphometric indices, FD is considered to be a characteristic parameter (19) of the difference between osteoporotic changes and normal bone density (20). Because osteoporosis induced by thalassemia has been detected in 30–50% of ß-TM patients (21), in the current study, the authors aimed to evaluate the cortical and trabecular mandibular bone morphology of children and adolescents with ß-TM and a systemically healthy group using FDA and different panoramic radiomorphometric indices.

The effects of many systemic diseases on the jaw with FD have been investigated in the literature (11,18). However, just two studies have been conducted in thalassemia patients (15,16).

Bayrak *et al*. (15) found a significant relationship between the TM and control groups regarding the mean FD (*p* = 0.014), contrary to the current study (*p* = 0.334). While Bayrak *et al*. (15) found the mean FD2 (the supracortical area above the mandibular angulus region) values, the mean FD4 (anterior side of the mental foramen) values were significantly lower in TM patients (*p* = 0.003 and *p* = 0.001, respectively). Meanwhile, Serindere and Belgin (16) did not find a significant relationship in any ROI (corpus ROI, angulus ROI, and interdental bone ROI) (*p* > 0.05). In the current study, when ROIs were taken into consideration, the mean FD values were not significantly different between regions in the ß-TM and control groups except for ROI 1 (*p* = 0.047). These different results between studies can be explained by the different ROI selection for each study.

Many studies showed that the MCW values of patients with an osteoporotic bone structure are lower than those of healthy control groups (22). Bayrak *et al*. (15) and Serindere and Belgin (16) found the mean MCW in the TM group to be 3.48 ± 1, 59, and 0.55, respectively, and in the control group to be 4.49 ± 1.09 and 1.00, respectively. In the current study, this value was 3.69 ± 0.81 and 4.96 ± 1.42 in the ß-TM group and control group, respectively. While the current study presents the mean MCW value similar to Bayrak *et al*.’s (15) results, it is higher than the results of Serindere and Belgin (16). These different results can be explained by the different age ranges of the patients participating in the studies. While Serindere and Belgin (16) conducted their studies in the TM group on ages 14–59 and in the control group on ages 13–77, Bayrak *et al*. (15) did not mention the age range in the material method part of their studies. The age range of the current study is 9–16 in both the ß-TM and control groups. Considering that the MCW decreases with age, it can be concluded that the age ranges of the two studies may be similar since the MCW values obtained in current study were similar to those of Bayrak *et al*. (15). In the present study, the mean MCW value was statistically lower in the ß-TM group than in the control group, similarly to the studies of Bayrak *et al*. (15) and Serindere and Belgin (16). Since all three studies show the same result, it can be concluded that the MCW is thinner in TM patients.

While in Bayrak *et al*.’ s (15) and Serindere and Belgin’s (16) studies, the mean PMI values were 0.44 ± 0.09 and 0.273, respectively, in the TM group, in the current study, this value was 0.24 ± 0.05. On the other hand, in Bayrak *et al*.’ s (15) and Serindere and Belgin’s (16) studies and the current study, the mean PMI values were 0.43 ± 0.15, 0.305, and 0.34 ± 0.1, respectively, in the control group. The PMI value in the study by Serindere and Belgin (16) was lower than the control group, which is similar to this study, whereas Bayrak *et al*. (15) found no significant differences between the TM and control groups.

The relationship between the mean MCW, the mean PMI values, and the mean FD was not examined in both studies (11,18) conducted on TM patients. In 2018, Kursun and Bayrak (18) found a correlation, which is consistent with the present study, between the mean MCW and FD values in type 1 diabetes mellitus patients (*p* = 0.011), but they found no such correlation, between the mean PMI and FD values (*p* = 0.7), which is inconsistent with the presented study. On the other hand, in 2016, Apolina ìrio *et al*. (11) did not find a correlation between the mean MCW and FD values in children with osteogenesis imperfecta. However, they found a correlation between the MCW and FD of the cortical bone, which is consistent with the presented study. The current study found a correlation between the MCW and ROI 1 FD (FD of the cortical bone) in the ß-TM and control groups. Moreover, this current study evaluated the same correlation between PMI-FD (*p* < 0.001), MCW-ROI 1 FD (*p* < 0.001), and PMI-ROI 1 FD (*p* < 0.001), both in the ß-TM and control groups. Therefore, the authors considered that this result might be a radiographic result. This is not just associated with ß-TM.

According to Ledgerton *et al*. (9), the MCI has excellent reliability among the radiomorphometric analyses, and it is a useful method for assessing bone quality. However, the MCI was insignificant between osteoporosis and healthy controls in some studies (9,23). In the presented study, contrary to Bayrak *et al*.’s study (15), there was a significant difference between the groups when the MCI is considered (*p* = 0.046).

A lower MCW can be correlated with the MCI, according to some studies (11,12,14). In the current study, while the mean MCW value correlated with the MCI in the control group (*p* = 0.04), the mean MCW value was not correlated with the MCI in the TM group (*p* = 0.493). Contrary to the current study, Apolina ìrio *et al*. (11) found a correlation between MCW measurements and the MCI (*p* = 0.001) in children with osteogenesis imperfecta.

The SVE is another radiomorphometric indices assessment for cortical bone. The SVE has not been evaluated in studies conducted on TM patients. In one study, the SVE was evaluated in a pediatric patient’s group, and a correlation was found between MCW measurements in the SVE in children with osteogenesis imperfecta (11). Consistent with the study mentioned, in the current study, there was a correlation between the MCW and the SVE in the ß-TM group (*p*= 0.031). The MCW values ranged between 2.5 and 2.8 mm in the "thin" group and between 3.5 and 3.7 mm in the "not thin" group in Apolina ìrio *et al*.’s (11) study. In this study, the mean MCW values were 3.25 ± 0.44 mm and 3.86 ± 0.86 mm in the "thin" and "not thin" groups, respectively. In the current study, the high MCW obtained compared to the mean of Apolonia ìrio *et al*.’s (11) study can be explained by the fact that osteogenesis imperfecta affects the bone structure more than ß-TM.

While Apolina ìrio *et al*. (11) found a correlation between the SVE and the FD, they found no correlation between the MCI and FD measures in any ROIs. In this study, no correlation was observed between any of the ROIs, the SVE, or the MCI in the ß-TM group, whereas a correlation was seen between ROI 4 and the SVE (*p* = 0.046) and between ROI 5 and the MCI in the control group (*p* = 0.022).

In the last few years, the FD has been used to examine the mandibular bone morphology of TM patients, and there are few studies on this subject. The results of the presented study contradict those of Bayrak *et al*. (15). While Bayrak *et al*. (15) found a significant relationship between the TM and control groups regarding the mean FD and angulus ROI FD, the current study did not find such a relationship. Moreover, Serindere and Belgin (16) did not find a significant relationship in the angulus ROI, similarly to the current study. The authors believe that the reasons for these different results should be investigated and that more studies with a larger sample size may show more accurate results. Sample size may be a limitation of the current study. In addition, occlusal force can affect mandibular bone structure. The occlusal situations of patients were not considered in the current study, because this research was a retrospective study. Also, the fact that the analyzed individuals had not been subjected to DEXA, which is the golden standard for evaluating BMD, is considered another limitation of the current study. On the other hand, as stated above, there are few studies on the mandibular bone morphology of TM patients and the current study contributes to the literature.

## Conclusions

Considering the results of the present study, while no difference was found between the trabecular bone morphology of ß-TM patients and the control group in children and adolescents, the mean cortical bone FD value was lower in the ß-TM group. While there was a significant difference between the ß-TM and control groups according to the MCI, there was no significant difference between the ß-TM and control groups according to the SVE. It was observed that only the MCW and PMI values of the ß-TM group were lower than the control group. The MCW, PMI, FD of cortical bone and MCI may be key indicators in individuals with beta-thalassemia major. Other results of the current study are as follows:

There was a significant relationship between the mean MCW, the mean PMI values, and the mean FD and mean ROI 1 FD values in both groups. The mean MCW values correlated with the SVE in both the control and ß-TM groups. The mean PMI values did not correlate with the SVE in the ß-TM group. Furthermore, the MCI values did not correlate with the MCW and PMI values in the ß-TM group. Moreover, the MCW-FD, PMI-FD, MCW-ROI 1 FD, and PMI-ROI 1 FD values were correlated in both groups.
